# Reply to: “Cytoreductive Surgery (CRS) and Hyperthermic Intraperitoneal Chemotherapy (HIPEC) Definitively Does Not Deserve Its Bad Reputation”

**DOI:** 10.1245/s10434-021-09743-z

**Published:** 2021-02-26

**Authors:** Philipp Horvath, Can Yurttas

**Affiliations:** 1 Department of General, Visceral and Transplant Surgery, Comprehensive Cancer Center, University of Tübingen, Tübingen, Germany; 2 National Center for Pleura and Peritoneum, University of Tübingen, 72076 Tübingen, Germany

It is with great interest that we read the letter to the Editor from Noiret et al. and the associated publication.^1^ We are very pleased to have the opportunity to frame an answer.

The results from Noiret et al.^1^ illustrate very impressively the impact of center experience (cut-off > 45 procedures/year) on postoperative mortality. Another important issue reported by the authors is that high-volume centers experienced increased major morbidity but had a significantly lower FTR index. On the other hand, the authors provided from their point of view several conceptual frailties of our previous study.^2^ The main difference between those two publications^1^,^2^ is the frame of the database. The data from our study were extracted from the nationwide German diagnosis-related group (DRG) statistics hosted by the German Federal Statistics Office. This is not a prospective database containing all the variables of interest in the context of PSM treatment. The database only contains clinical information necessary for DRG- and cost-refund calculations. Noiret et al. are absolutely right that, in our publication, several highly interesting parameters (PCI scores, CC scores, degree of experience with PSM treatment, and the respective case load per year) were missing. This was unfortunately due to the frame of the database. On the other hand, the prospective database (PSMI) seemed to provide a lot more clinically relevant information, and therefore Noiret et al. answered a variety of important questions in the context of PSM treatment, which could not be addressed by us.

Our conclusion that the reduction of the FTR index was due to a higher degree of centralization of PSM treatment was an assumption underlined by the fact that CRS and HIPEC procedures in Germany should only be performed in centers of excellence or competence, and from 2009 to 2018, a lot of centers were certified. The Deutsche Gesellschaft für Allgemein- und Viszeralchirurgie (DGAV, German Association of General and Visceral Surgery) provides a certificate only to centers that fulfill certain requirements (i.e., case load/year).

Overall, we are absolutely in line with the conclusion from Noiret et al. that these publications reporting on almost 16,000 patients should redefine the view on CRS and HIPEC procedures. Hopefully, we will see a decrease in the reluctance of some physicians to refer patients to high-volume centers for evaluation of CRS and HIPEC.
